# Micrometer-Sized Titanium Particles Induce Aseptic Loosening in Rabbit Knee

**DOI:** 10.1155/2018/5410875

**Published:** 2018-02-13

**Authors:** Hao Xu, Cui-cui Guo, Zheng-yu Gao, Chang-yao Wang, Hai-ning Zhang, Cheng-yu Lv, Zi-yan Yin, Ying-zhen Wang

**Affiliations:** ^1^Department of Joint Surgery, The Affiliated Hospital of Qingdao University, Qingdao, Shandong 266000, China; ^2^Department of Sports Medicine, The Affiliated Hospital of Qingdao University, Qingdao, Shandong 266000, China; ^3^Department of Rehabilitation Medicine, The Affiliated Hospital of Qingdao University, Qingdao, Shandong 266000, China; ^4^Biostatistics Department, Medical College of Wisconsin, Milwaukee, WI, 53226, USA

## Abstract

Wear debris induced aseptic loosening is the leading cause of total knee arthroplasty (TKA) failure. The complex mechanism of aseptic loosening has been a major issue for introducing effective prevention and treatment methods, so a simplified yet efficient rabbit model was established to address this concern with the use of micrometer-sized titanium particles. 20 New Zealand white rabbits were selected and divided into two groups (control = 10, study = 10). A TKA surgery was then performed for each of them, with implantation of a titanium rod prosthesis which was coated evenly with micrometer-sized titanium in the study group and nothing in the control group, into right femoral medullary cavity. After 12 weeks, all the animals were euthanized and X-ray analyses, H&E staining, Goldner Masson trichrome staining, Von Kossa staining, PCR, and Western blotting of some specific mRNAs and proteins in the interface membrane tissues around the prosthesis were carried out. The implantation of a titanium rod prosthesis coated with 20 *μ*m titanium particles into the femoral medullary cavity of rabbits caused continuous titanium particle stimulation around the prosthesis, effectively inducing osteolysis and aseptic loosening. Titanium particle-induced macrophages produce multiple inflammatory factors able to activate osteoclast differentiation through the OPG/RANKL/RANK signaling pathway, resulting in osteolysis while suppressing the function of osteoblasts and reducing bone ingrowth around the prosthesis. This model simulated the implantation and loosening process of an artificial prosthesis, which is an ideal etiological model to study the aseptic prosthetic loosening.

## 1. Introduction

In the past 40 years, total knee arthroplasty (TKA) is one of the most successful surgeries worldwide [1]. However, as its use has extended over time, avoiding the problem of prosthetic failure has become increasingly difficult [2, 3]. Approximately 10–15% of TKAs failed within 15 years after the primary knee replacement surgery and approximately 70% of these failures were caused by aseptic loosening [4]. This situation will continue to deteriorate as society ages, leading to heavy psychological, physical, and economic burdens on patients [5–7]. The mechanism of prosthetic aseptic loosening after TKA is complex. Debris produced at various prosthetic interfaces may trigger local inflammatory reactions [8], and the fixation pattern of the prosthesis may produce stress-shielding effects, resulting in local mechanical imbalance [9]. Both of these issues can result in bone resorption and osteolysis around the prosthesis, which may eventually lead to prosthetic loosening. Although the causes of bone dissolution and its detailed pathological process remain to be elucidated, the biological process of particle-induced osteolysis has emerged as a consensus within the medical community [10]. Upon this theoretical basis, we can modulate the key aspect of aseptic loosening in animal models and, thereby, develop a platform for further studies of the prevention and treatment of prosthetic aseptic loosening after TKA.

The optimal animal model should accurately simulate the whole process of aseptic loosening induced by wear particles, although economic and practical factors must also be considered. Small animal models such as murine skull models are rapid, economical, and repeatable [11]; however, these models lack the implantation of simulated prostheses, which may better mimic the actual acute osteolysis process, and thus the mechanical fretting and local responses at the contact interface between the prosthesis and surrounding bone tissue cannot be simulated [12]. Although large animal models with real prosthesis implantation are ideal for the simulation of the mechanical loosening process of implanted prostheses, such models are difficult to implement on a large scale because of the high associated costs and decreased maneuverability associated with the highly technical operation required [13–15]. Rabbits are moderately sized animals and are widely used in experiments. Thus, they are expected to be ideal for the development of an appropriate model because the real arthroplasty surgery can be implemented by implanting the prosthesis into the knee joint, resulting in lower costs and increased maneuverability [16].

The typical wear-and-tear materials chosen for this type of study include polymer polyethylene, cobalt chromium molybdenum alloy, and titanium alloy [17]. Polyethylene and Co-Cr-Mo alloy wear particles are relatively difficult to obtain from loosened prostheses, and thus these types of materials are impractical for a wide range of zoological research. Titanium particles with diameters less than 20 *μ*m are relatively easy to prepare, and their biological effects do not differ substantially from those of other abrasive particles [18]. Therefore, we conducted this study using micrometer-sized titanium particles to establish a prosthetic loosening knee model in rabbit and aimed to verify the efficiency of this model for simulating the process of aseptic loosening and exploring its underlying mechanism.

## 2. Materials and Methods

### 2.1. Animals and Materials

(1) Twenty healthy adult female New Zealand white rabbits were randomly divided into the study group and control group (10 animals per group). The rabbits were purchased from Shandong Lukang Medicine Corporation (Shandong province, China). The average weight was 2.85 kg. All animal procedures were inspected and approved by the Ethic and Animal Welfare Committee of the Affiliated Hospital of Qingdao University, confirming that all experiments were performed in accordance with relevant guidelines and regulations (Approval Number: N02165782).

(2) Titanium rod prostheses were purchased from Stryker Co. Ltd., USA, and had the following characteristics: shape: smooth cylinder; length: 2.0 cm; and diameter: 0.5 cm. The prostheses were sterilized with high-pressure steam before use.

(3) Titanium particles were purchased from Alfa Aesar Chemical Co. Ltd. (China). The diameters of all particles were less than 20 *μ*m.

(4) The Animal Laboratory of the Affiliated Hospital of Qingdao University supplied the special animal feeding cages and animal experimental operation systems.

### 2.2. Animal Surgery

**Figure 1:**
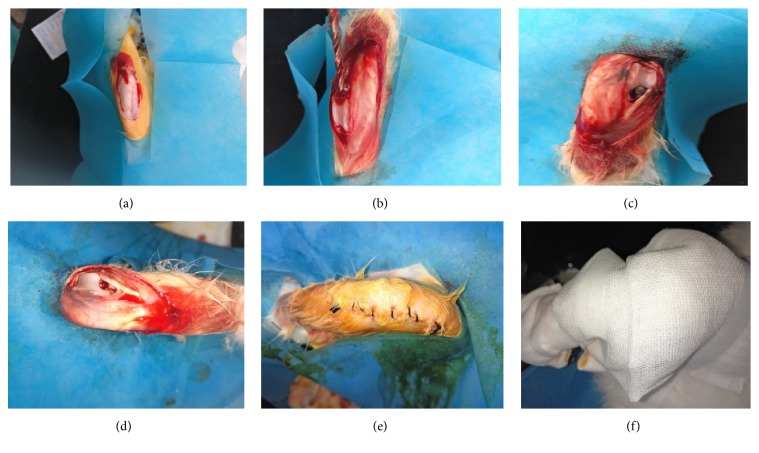
Operative process of titanium rod prosthesis implantation surgery. (a) Skin incision and exposure of the knee joint. (b) We opened the femoral medullary cavity and expanded the medullary cavity with increasing sizes of drills. ((c) and (d)) Suitable size of titanium rod prosthesis was implanted into the medullary cavity. (e) The incision was closed after thorough rinsing. (f) The lower limb was dressed without additional fixation.

One week before surgery, all rabbits were provided with adaptive feed, and the right knee was chosen for operation. Each rabbit was anesthetized with an intraperitoneal injection of xylazine hydrochloride (0.2 ml/kg) and fixed on a special surgical scaffold in a supine position. After skin preparation, the operative knee joint portion was sterilized with 2.5% povidone-iodine and an aseptic hole towel was laid over the surgical area. An anterior middle skin incision and medial parapatellar approach were adopted to expose the subcutaneous tissue, joint capsule, and articular cartilage surface layer by layer. We opened the femoral medullary cavity with drills just above the endpoint of the posterior cruciate ligament in the femoral intercondylar fossa and expanded the medullary cavity with increasing sizes of drills along the femoral longitudinal axis from 0.3 cm to 0.5 cm. Then, we implanted the selective titanium rod prosthesis into the medullary cavity at a depth of more than 2 cm. The incision was closed after thorough rinsing, and the lower limb was dressed without additional fixation. The surface of the titanium rod prosthesis implanted in the study group was evenly coated with approximately 50 *μ*g of titanium particles before implantation, whereas the titanium rod prosthesis used in the control group was uncoated (Figure 1).

### 2.3. Postoperative Management

In addition to strict sterilization during the operation, all animals were treated with penicillin intramuscularly at a dose of 4 million units given 2 times per day for 3 days to prevent infection. All animals were allowed to move freely without any fixation of the lower limbs.

### 2.4. Animal Sacrifice and Tissue Harvest

All animals were euthanized 12 weeks after the operation. The synovial tissue of the ipsilateral knee was harvested and processed in zinc formalin (10%) for paraffin sectioning. The right distal femur with the titanium rod was harvested and dissected with a hammer 2 cm away from the knee joint surface. The distal part was preserved in freezer at −80°C and prepared for hard-tissue sectioning. We retained two pieces (approximately 50 *μ*g) of residual interface membrane tissue collected from around the titanium rod at the proximal part of the femur: one immersed in RNAlater® Solution and the other stored in a 1.5 ml tube and sealed quickly with liquid nitrogen. Both were preserved in a −80°C freezer for real-time polymerase chain reaction (PCR) and Western blotting.

### 2.5. Radiological Evaluation

Anterior-posterior (AP) and lateral X-ray films of the right knee were taken with mobile C-arm X-ray machine (SIREMOBIL Compact L, Siemens Ltd., Germany), 2 days before sacrificing to find signs of osteolysis and prosthesis loosening.

### 2.6. Bone and Synovial Tissue Staining and Analysis

The synovial tissue of the knee was subjected to paraffin sectioning and stained with hematoxylin and eosin (H&E). Bone tissues containing titanium rod prostheses were subjected to hard-tissue sectioning without decalcification. Grinding of the nondecalcified bone tissue preserved the structure of the trabecular bone well and accurately reflected the situation of bone growth and mineralization, facilitating the investigation of bone integration and absorption. The bone tissue was preformed into 1 cm sections in advance and was cut into noncontinuous sections of 50 microns each along the cross section of the femur using a hard-tissue microtome (EXAKT 300CP, Germany). The primary section was thinner with graded SiC&Al_2_O_3_ sandpaper and polished with fine sandpaper at last. The final thickness of hard-tissue section was 5 *μ*m. The hard-tissue sectioning was performed using Goldner's trichrome staining and Von Kossa staining. The bone-prosthesis contact rate (B-PCR) and bone volume percentage (BVP) were calculated based on Goldner's trichrome staining as follows.

B-PCR (%) = the length of the circumference of the bone in contact with the prosthesis/total circumference of the prosthesis [19].

BVP (%) = squares of the bone tissue in a 1 mm area around the prosthesis/total squares of the 1 mm area around the prosthesis [20].

Von Kossa staining can be used to measure the amount and average area of calcium salt deposit islands (CSDIs) of cortical bone to determine the bone mass around the prosthesis [21, 22].

### 2.7. Western Blotting

The stored tissues were ground in liquid nitrogen and then homogenized in radioimmunoprecipitation assay (RIPA) buffer (400 *μ*L) containing protease and phosphatase inhibitors at 4°C. Total protein was collected after centrifugation at 12,000 rpm/min for 10 min and quantified by a BCA™ Protein Quantification Kit. The protein extracts were used for Western blotting. Equal amounts of total protein were separated by 12% sodium dodecyl sulfate polyacrylamide gel electrophoresis (SDS-PAGE) and transferred electrophoretically onto a polyvinylidenedifluoride (PVDF) membrane. The membrane was blocked in Tris-buffered saline containing 0.05% (v/v) Tween-20 (TBST) with 5% (w/v) bovine serum albumin (BSA) for one hour and probed overnight at 4°C with appropriate mouse monoclonal anti-osteocalcin (OCN) (1 : 1000 dilution) and anti-receptor activator of nuclear factor kappa-B (NF-*κ*B) ligand (RANKL), polyclonal rabbit anti-tryptophan-regulated attenuation protein (TRAP) (1 : 1000 dilution) and anti-osteoprotegerin (OPG) (1 : 1000 dilution), and *β*-actin (1 : 1000 dilution). After washing in TBST, the membrane was incubated with horseradish peroxidase (HRP) conjugated with anti-mouse and anti-rabbit-immunoglobulin G (IgG) secondary antibody (1 : 2000 dilution) at 37°C for two hours. After washing in TBST again, the protein bands were detected using a chemiluminescence detection system. Densitometric analysis was performed using ImageJ 1.41 (National Institutes of Health, Bethesda, MD, USA).

### 2.8. Real-Time PCR

Real-time reverse-transcription PCR (RT-PCR) was performed to assess the state of local bone formation and resorption after the implantation of the titanium rod prosthesis. Total RNA from interface membrane tissues around the prosthesis was extracted in TRIzol reagent (Invitrogen). cDNA was obtained by reverse transcription from 0.5 *μ*g of total RNA. First, 12 *μ*l of a reaction mixture was composed of denatured RNA; 10 mM each of the nucleotide triphosphates (dNTPs) and 50 *μ*M Oligo(DT)_20_ primer were incubated at 65°C for 50 min and then placed on ice. Then, 8 *μ*l of the master reaction mix containing 5xcDNA synthesis buffer, 0.1 M dithiothreitol (DTT), 40 U/ml RNaseOUT, and 15 units/*μ*l ThermoScript reverse transcriptase were added. The reaction mixture was incubated in a DNA Thermal Cycler (Perkin Elmer, CT) at 55°C for 50 min, followed by 95°C for 5 min. All of the primers were designed using Primer3 (v.0.4.0 http://frodo.wi.mit.edu/primer3). Using the Ct value of *β*-actin as an internal control, the gene expressions of OCN, OPG, RANKL, and TRAP5b in the study group samples were quantitated and compared to the results of the control group according to the formula provided by the manufacturer (PE Applied Biosystems).

### 2.9. Statistical Analysis

Image and data processing were performed using Image-Pro Plus 6.0 (IPP 6.0, Media Cybernetics, Inc., USA). Statistical analyses were conducted using GraphPad Prism 6.03 (GraphPad Software La Jolla, CA). Mean and standard deviation were provided for numeric outcomes. The impact of our model was assessed using *t*-test and one-way analysis of variance (ANOVA), with a *p* value of 0.05 or less being considered as an indicator of statistical significance.

## 3. Results

### 3.1. Titanium Particles Induced Osteolysis around Prosthesis in Radiological Examination

**Figure 2:**
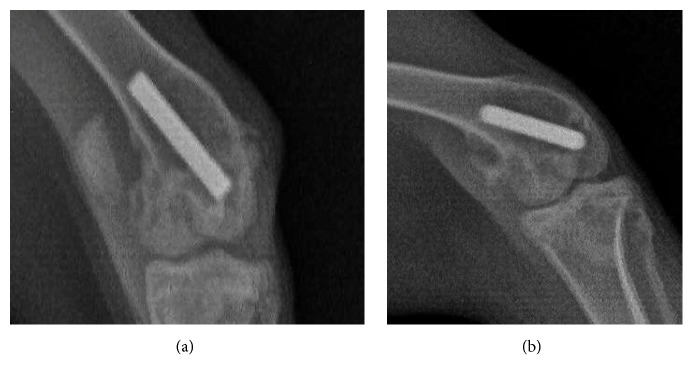
X-ray photos before harvest. (a) No obvious signs of osteolysis were found in the control group. (b) Clear signs of osteolysis around the titanium rod could be detected in the study group.

After 12 weeks of feeding, we collected radiological photos of both groups 2 days before sacrificing them. In both groups, all titanium rod prostheses were implanted correctly into the femoral medullary cavities of the chosen knees. Compared with the control group, significant radiological signs of osteolysis around prosthesis were observed in the study group by X-ray (Figure 2).

### 3.2. Titanium Particles Triggered Obvious Inflammatory Reactions in the Synovial Tissue

**Figure 3:**
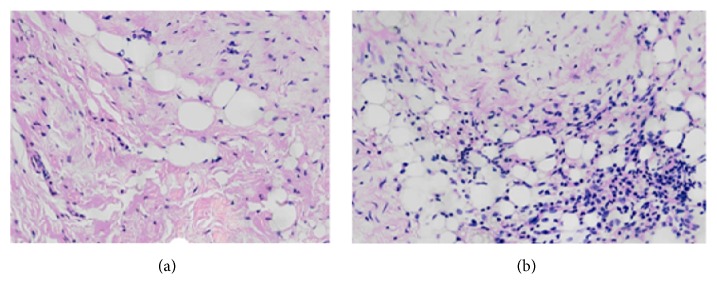
Results of H&E staining of synovial tissue (100x, bar = 100 *μ*). (a) No obvious infiltration of inflammatory cells and fewer plicae were found in the synovial tissue of the control group. (b) There were abundant inflammatory cells infiltrating the synovium in the study group, combined with the proliferation of fibrous connective tissue.

H&E staining revealed that the micrometer-sized titanium particles on the surface of the prosthesis could stimulate a significant inflammatory response. Abundant inflammatory cells infiltrating the synovium and obvious synovial hyperplasia with larger plicae were observed in the study group, combined with the proliferation of fibrous connective tissue. By contrast, no apparent infiltration of inflammatory cells and fewer plicae were found in the synovial tissue of the control group (Figure 3).

### 3.3. Titanium Particles Impacted Osteointegration between Bone and Prosthesis and Significantly Reduced Periprosthetic Mineralization

**Figure 4:**
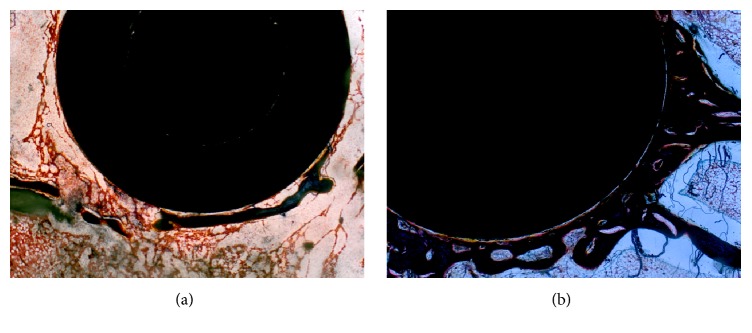
Results of Goldner's Masson trichrome stains (40x, bar = 250 *μ*m). (a) The integration of the bone and prosthesis was better in the control group. (b) Worse integration of bone and prosthesis and a larger gap could be detected around the prosthesis in the study group.

**Figure 5:**
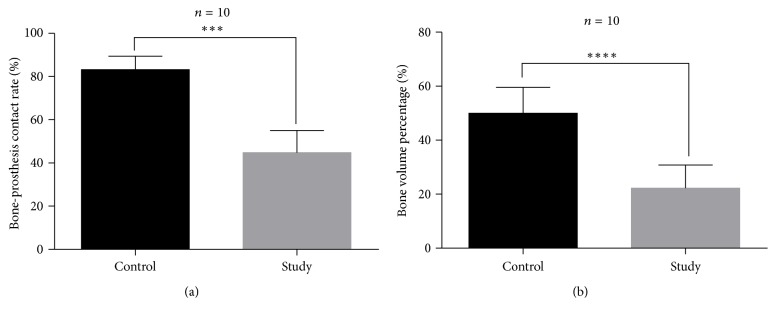
Comparison of B-PCR and BVP between the two groups. (a) Titanium particles reduced the B-PCR significantly (*p* < 0.001). (b) The BVP of the study group was significantly lower (*p* < 0.001). ^*∗∗∗*^*p* < 0.001; ^*∗∗∗∗*^*p* < 0.0001.

The hard-tissue sections of bone including the titanium rod prosthesis were subjected to Goldner's trichrome staining and Von Kossa staining. Goldner's trichrome staining indicated worse integration of bone and prosthesis and a larger gap around the prosthesis in the study group. The area surrounding the prosthesis was mainly filled with fibrous connective tissue. By contrast, the integration between the bone and prosthesis was much better in the control group. Indeed, bone tissue was distributed evenly around the prostheses without obvious fibrous connective tissue proliferation (Figure 4). According to the results of Goldner's staining and image analysis using IPP 6.0, both the B-PCR and BVP of the study group were significantly lower than those of the control group (*p* < 0.05, Figure 5), which indicated lower contact ratio between bone and prosthesis and worse bone formation around the prosthesis.

**Figure 6:**
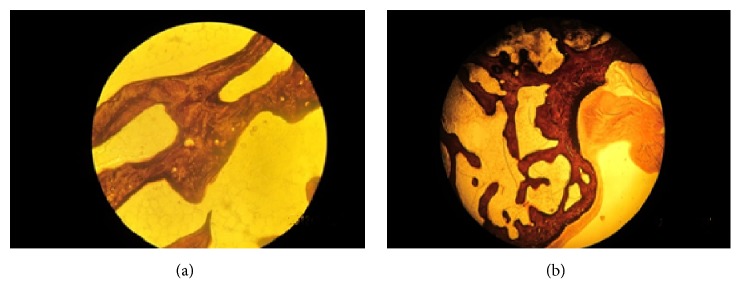
Results of Von Kossa staining (100x, bar = 100 *μ*m). (a) The calcium deposit islands around the prosthesis in the control group were mostly star-shaped and less abundant but larger. (b) The calcium deposit islands in the study group were mainly linear and more abundant but smaller in size.

**Figure 7:**
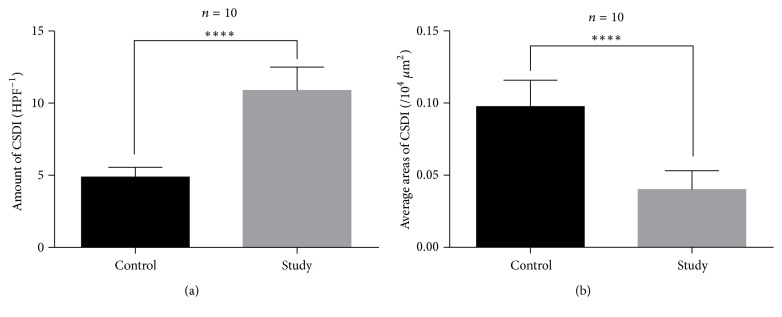
The results of CSDI indicate that the bone mineralization capacity was lower due to titanium particles. (a) Comparison of the amount of CSDI (*p* < 0.001). (b) Comparison of the average areas of CSDI (*p* < 0.001). ^*∗∗∗∗*^*p* < 0.0001.

Based on the Von Kossa staining results, we used IPP 6.0 to calculate the amount and area of CSDIs around the prostheses in the two groups. The calcium deposit islands in the study group were mainly linear and were more abundant but smaller in size (Figure 6). In contrast, the calcium deposit islands around the prosthesis in the control group were mostly star-shaped and were less abundant but larger. These results indicated that the bone mineralization capacity of the study group was significantly lower than that of the control group; this difference was statistically significant (Figure 7). In general, a decrease in bone mineralization suggests inhibition of the osteoblast function and reduced bone formation.

### 3.4. Titanium Particles Induced Osteoclastic Osteolysis and Inhibited the Function of Osteoblast around Prosthesis

**Figure 8:**
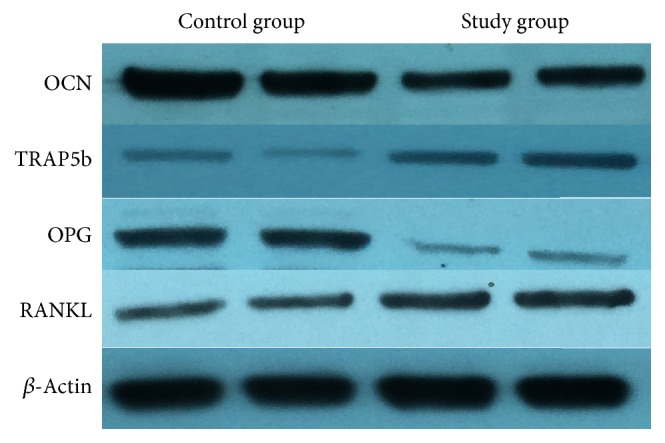
Results of Western blotting of relative proteins. Compared with the control group, the OCN content in the study group significantly decreased, whereas the TRAP protein content in the study group increased. In contrast, the OPG protein content significantly decreased in the study group, whereas the RANKL protein content increased.

**Figure 9:**
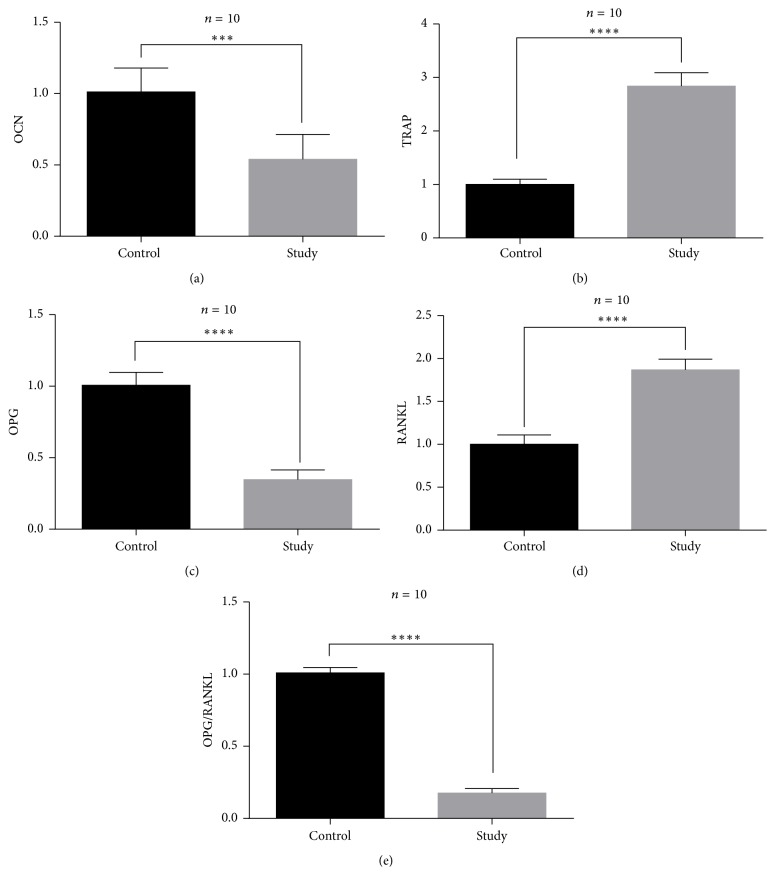
Results of real-time PCR of relative mRNAs. (a) mRNA content of OCN decreased in the study group (*p* < 0.001). (b) mRNA content of TRAP increased in the study group (*p* < 0.001). (c) mRNA content of OPG decreased in the study group (*p* < 0.001). (d) mRNA content of RANKL increased in the study group (*p* < 0.001). (e) The OPG/RANKL ratio significantly decreased in the study group (*p* < 0.001). ^*∗∗∗*^*p* < 0.001; ^*∗∗∗∗*^*p* < 0.0001.

Real-time PCR and Western blotting were performed to determine the contents of specific mRNAs and proteins in the interface membrane tissues around the prostheses. Two pairs of samples were processed simultaneously. The appropriate exposure time and developer were selected based on *β*-actin. Compared with the control group, the protein content of OCN, specific marker of osteoblast, was significantly decreased in the study group (*p* < 0.01), whereas the protein content of TRAP5b, specific marker of osteoclast, was increased in the study group (*p* < 0.01). Meanwhile, the OPG protein content significantly decreased in the study group, whereas the RANKL protein content increased. As a result, the OPG/RANKL ratio decreased significantly. The OPG/RANKL balance is key to maintaining balanced bone metabolism. Decreased OPG/RANKL ratio enhanced activity of osteoclast, which also led to osteoclastic osteolysis. From the above results, we concluded that titanium particles existing around the prosthesis induced osteoclastic osteolysis and inhibited the function of osteoblast around prosthesis. The mRNA contents corresponding to these proteins exhibited the same trends as demonstrated by Western blotting, as determined via real-time PCR (Figures 8 and 9).

## 4. Discussion

Our model has several interesting advantages. (1) This model could simulate the whole process of aseptic loosening relatively accurately and reveal almost all characteristics of the main pathological process of aseptic loosening. Thus, it is an appropriate etiological model for studying the mechanism and development of aseptic loosening after knee arthroplasty. (2) The model successfully simulated the particle removal process. In this model, a variety of experimental conditions could be created in the living body, and real prosthesis implantation was performed to obtain an effective interface membrane tissue containing wear particles around the prosthesis. (3) This model allows researchers to work with a living body. Indeed, studying the mechanism of aseptic loosening on the pathological and histopathological levels in a living body with true implanted prostheses in their original locations is more authentic. More importantly, this model could be utilized in biomechanical studies of aseptic loosening. (4) This method of evenly coating the surface of the titanium rod prosthesis with micrometer-sized titanium particles was easy to operate and diminished the resulting error.

In this study, we used nondecalcified hard-tissue sections to observe the local histopathological changes involved in wear debris induced prosthetic loosening to investigate the relative metabolic changes around the prosthesis relatively intuitively and authentically. Although no significant differences in osteolysis and prosthetic loosening were observed between the two groups upon radiological examination, the results of Goldner's staining of the hard-tissue sections revealed that the B-PCR and BVP of the study group were significantly lower than those of the control group. The bone loss around the prosthesis was more serious in the study group, and the residual gaps were filled with fibrous tissues, resulting in instability of the implanted prosthesis prosthetic and, finally, loosening. As shown by Von Kossa staining, the number of calcium deposit islands in the study group was much higher and mostly exhibited a cord-like shape. In contrast, the average area of the calcium deposit islands in the control group was larger, and most islands exhibited map-like shapes, indicating that osteogenesis around the prosthesis was significantly inhibited by titanium particles. These results reflect increased osteolysis and decreased bone formation in periprosthetic manner. We also detected an obvious inflammatory reaction in the synovitis tissue via H&E staining and attributed this reaction to the titanium particles around the prosthesis. The variety of inflammatory factors derived from the inflammatory response should promote osteolysis and inhibit bone formation, eventually leading to aseptic loosening [23]. Therefore, micrometer-sized titanium particle-induced rabbit model showed the basic biological and histological characteristics of aseptic loosening.

The biological processes resulting from multiple factors induced by wear debris constituted the main mechanism leading to aseptic loosening of the prosthesis. First, wear debris induced localized macrophages to produce IL-6, IL-1, TNF-*α*, prostaglandin E2 (PGE2), and other inflammatory factors, the release of which could stimulate the activation of osteoclasts, leading to a secondary osteolytic reaction and prosthetic aseptic loosening [24, 25], as confirmed by the interface membrane around the loosened prosthesis. The interface membranes were mainly composed of macrophages, fibroblasts, osteoblasts, osteoclasts, other cells, and abundant wear particles, reflecting a nonspecific inflammatory response of the bone to wear particles from the prosthesis [26]. In their experimental study, Ren et al. [27] injected polyethylene particles continuously into the femoral tube and fluorescent-labeled macrophages into another location. The results of immunohistochemistry microcomputed tomography (CT) and cytological analysis revealed that the number of macrophages in the study group that received polyethylene particle injections was much higher than that in the control group. Additionally, macrophages migrated toward the perfusion site. The bone density of the study group was lower than that of the control group, as determined by labeling the bone remodeling process, which confirmed that the polyethylene particles induced the activation of macrophages and caused osteolysis. Other studies have also demonstrated that wear particles can cause apoptosis in macrophages and multinucleated giant cells around prostheses [28]. Landgraeber et al. [29] determined the concentration of senescence-associated beta-galactosidase (SA-B-Gal), a specific marker of senescence in macrophages, and found that the degree of cell senescence was significantly higher in the joint surface and joint capsule of patients with aseptic loosening. After further statistical analysis, the expression of SA-B-Gal on the surfaces of macrophages and multinucleated giant cells was determined to be highly correlated with the amount of wear particles. In our study, H&E staining revealed significant inflammatory cell infiltration in the synovial tissue from the knee in the study group, confirming that wear particles could induce a local inflammatory response.

The local effects of particles and the release of various inflammatory factors could stimulate osteoclast activation, resulting in a significant osteolytic response. Wear debris particles typically stimulate osteoclast proliferation and differentiation by activating the OPG/RANKL/RANK pathway [30]. Hartmann et al. [31] examined the mRNA expression of RANK and OPG and the binding activity of NF-*κ*B by coculturing titanium particles with IC-21 macrophages. The results showed that the osteolytic reaction process could be initiated by titanium particles through the OPG/RANKL/RANK pathway. The activation of osteoclasts was attributable to the specific binding of the expressed macrophage colony-stimulating factor (M-CSF) and NF-activated receptor ligands (RANKL) and to the colony-stimulating factor 1 receptor (c-fms) and RANK on osteoclast precursor cells. Specific binding and the activity of osteoclasts can also be maintained through this pathway. Specifically, RANK is type I of transmembrane protein located on the surface of osteoclasts and precursor cells that specifically binds to RANKL to activate the OPG/RANKL/RANK signal transduction system, thereby promoting osteoclast differentiation and maturation. The differentiation of mature osteoclasts could further express protooncogene fos (c-fos), protooncogene tyrosine-protein kinase Src (c-src), cathepsin K (CATK), TRAP, calcitonin receptor (CTR), and other osteolysis-related factors [32]. Additionally, osteoblasts expressed a certain amount of RANKL to promote the differentiation of osteoclasts but secreted the corresponding amount of OPG to prevent the excessive absorption of bone [33]. The real-time PCR results found here showed that the OPG mRNA content around the titanium rods was significantly decreased, whereas that of RANKL was upregulated in the study group. The Western blotting results revealed the same trend of related protein quantification. Our study also confirmed that wear debris coated on a prosthesis could induce an imbalance in the OPG/RANKL ratio, leading to osteolysis around the prosthesis and, eventually, the development of aseptic loosening through the OPG/RANKL/RANK signaling pathway.

The micrometer-sized wear particles not only induced osteoclastic osteolysis but also inhibited the osteoblast function around the prosthesis to reduce the bone formation and, thus, accelerate the osteolysis process. Pioletti et al. [34, 35] found that wear particles can decrease the reproductive capacity to inhibit the osteoblast function and can also reduce osteoblast numbers by triggering apoptosis in osteoblasts. Other studies on mouse skull models determined that titanium particles can substantially affect the activity of skull osteoblasts and that some osteoblasts become toxic to other osteoblasts after engulfing titanium particles, eventually inducing apoptosis in other osteoblasts [19]. Fujii et al. [20] found that wear particles could stimulate the expression of cytokines on the surface of fibroblasts and osteoblast cells, such as IL-6, IL-1*β*, TNF-*α*, and cyclooxygenase- (COX-) 2, which plays key roles in the osteolysis process. Maoqiang et al. [21] also demonstrated that titanium particles could inactivate T cells (nuclear factor of activated T cells 1 [NFATc1]) through the 11R-VIVIT peptide, which was essential for the activation of RANK. The final result was that the differentiation of osteoblasts and the mineralization of RC cells, a cell culture model composed of many osteogenic precursor cells, were affected. These findings were confirmed in our study. We observed that both the mRNA and protein contents of OCN, a specific marker of osteoblast differentiation, decreased significantly, indicating that the differentiation of osteoblasts into the mineralization phase decreased.

Briefly, the mechanism underlying wear particle-induced aseptic loosening in this rabbit model primarily involved two processes. First, the wear particles induced the activation of macrophages, which released a variety of inflammatory mediators to activate osteoclast-induced osteolysis. Second, the osteoblastic activity was suppressed to reduce bone formation. The combination of these two processes caused osteolysis around the prosthesis and eventually led to aseptic loosening. In this process, the OPG/RANKL/RANK signaling pathway was particularly important.

In this study, we chose the New Zealand white rabbit as the study model because of its easy feeding and low price. The developed model was able to simulate the most important pathological features of the aseptic loosening process, and thus it is a good etiological model for investigating aseptic prosthetic loosening. This rabbit model allows various experimental conditions to be integrated into the prosthesis in a living body, creating an effective aseptic loosening state with wear particles around the prosthesis. This state was consistent with the clearance mechanism of the body's response to wear particles. In this living model, we could impose different interventions on the implanted prosthesis to study the mechanism of aseptic loosening in terms of its organization and impacts on cells and molecules. This model is especially suitable for simulated living body research relating to prevention and treatment of aseptic loosening.

However, the model had some shortcomings. Although this model mainly modulated the aseptic loosening process of the knee, it was not exactly the same as the real knee prosthesis in the clinic, and thus it may be biased relative to the real situation of aseptic loosening, especially in terms of the mechanical mechanism. Although the periprosthetic osteolysis and aseptic loosening were observed pathologically, we were unable to acquire radiological evidence of aseptic loosening after 12 weeks of feeding. Some authors, such as Pap et al. [22], have modified similar models to increase the daily and weekly activity and thereby create mechanical loading conditions. Although the final results showed effective interface membrane formation around the prosthesis, no typical radiological images or symptoms of aseptic loosening were found. The relatively long period of feeding required might be a limitation for future large-scale rapid research. Therefore, improving the efficiency and success rate of this model should be pursued.

## 5. Conclusion

In conclusion, a rabbit model of aseptic loosening of the knee joint was successfully established by implanting a titanium rod prosthesis coated with 20 *μ*m micrometer-sized titanium particles. The main mechanism underlying this aseptic loosening model was that titanium particles induced osteoclast activation and osteoblast dysfunction through the OPG/RANKL/RANK signaling pathway, eventually leading to periprosthetic osteolysis. This model is an ideal etiological and biomechanics model for investigating prosthesis aseptic loosening that is economical, effective, rapid, and reproducible. This model could also be used in research relating to the prevention and treatment of aseptic loosening.
